# Short-Term Impact of Traffic-Related Particulate Matter and Noise Exposure on Cardiac Function

**DOI:** 10.3390/ijerph17041220

**Published:** 2020-02-13

**Authors:** Jean Marie Buregeya, Philippe Apparicio, Jérémy Gelb

**Affiliations:** Centre Urbanisation Culture Société, Institut National de la Recherche Scientifique, Montréal, QC H2X 1E3, Canada; jean-marie.buregeya@ucs.inrs.ca (J.M.B.); jeremy.gelb@ucs.inrs.ca (J.G.)

**Keywords:** cycling, heart rate variability, air pollution, environment noise, PM_2.5_, traffic-related air pollution, traffic noise

## Abstract

Exposure to traffic-related air pollution and noise exposure contributes to detrimental effects on cardiac function, but the underlying short-term effects related to their simultaneous personal exposure remain uncertain. The aim is to assess the impact of total inhaled dose of particulate matter and total noise exposure on the variations of electrocardiogram (ECG) parameters between pre-cycling and post-cycling periods. Mid-June 2019, we collected four participants’ personal exposure data related to traffic-related noise and particulate matter (PM_2.5_ and PM_10_) as well as ECG parameters. Several Bayesian linear models were built to examine a potential association between air pollutants and noise exposure and ECG parameters: heart rate (HR), standard deviation of the normal-to-normal intervals (SDNN), percentage of successive RR intervals that differ by more than 50 ms (pNN50), root mean square of successive RR interval differences (rMSSD), low-frequency power (LF), high-frequency power (HF), and ratio of low- to high-frequency power (LF/HF). We analyzed in total 255 5-min segments of RR intervals. We observed that per 1 µg increase in cumulative inhaled dose of PM_2.5_ was associated with 0.48 (95% CI: 0.22; 15.61) increase in variation of the heart rate, while one percent of total noise dose was associated with 0.49 (95% CI: 0.17; 0.83) increase in variation of heart rate between corresponding periods. Personal noise exposure was no longer significant once the PM_2.5_ was introduced in the whole model, whilst coefficients of the latter that were significant previously remained unchanged. Short-term exposure to traffic-related air and noise pollution did not, however, have an impact on heart rate variability.

## 1. Introduction

Traffic-related air and noise pollution exposure is recognized as one of the major public health challenges of the 21st century. It is well-known that exposure to air and noise pollution contributes to detrimental effects on respiratory and cardiovascular systems [1,2,3]. These exposures are indeed a major contributor to increased length of in-hospital stay, morbidity, and mortality associated with chronic diseases [4,5,6]. Although the causes are multifactorial, one of the pathophysiological mechanisms entails acute changes in cardiovascular autonomous modulation [7]. Various studies have also shown that the autonomic nervous system regulates different functions of the body, such as breathing, gas exchange, blood pressure, heart muscles, etc., and thus allows the maintenance of the internal homeostasis of the organism [8,9]. Heart rate variability can hence be used to measure short-term health effects, thereby showcasing how air and noise pollution disrupts the autonomous cardiac function [2,10]. While both air and noise pollution co-occur in the same environment and alter the cardiac autonomic function, their health endpoint resemblance may reflect either their interaction or one acting as a confounding/effect modifier on the outcome [3].

To reduce the impact of traffic-related air and noise pollution exposure, different authors promote healthy living and consistently advocate a shift from automobiles to active transportation, such as cycling or walking [11,12,13]. In urban environments, however, cyclists may still be at increasing risks inasmuch as they have a higher ventilation rate of pollutants, which increases the inhaled dose of air pollutants [14,15]. For instance, a recent study has shown that during rush hours in Montreal, cyclists had 3.7 times higher inhaled doses of nitrogen dioxide than motorists, and they were also exposed to higher levels of noise (1.9 dB(A)) [14]. To this end, several studies have found an association between exposure to traffic-related air pollution [16,17,18] and exposure to noise [2,3,19,20] and their personal impact on heart function. To our best knowledge, there is nonetheless a scarcity of studies in the scientific literature that explore whether and how the simultaneous traffic-related air and noise pollution exposure disrupts the heart function in real-world settings. Thus, the study objective was to examine in real-life setting short-term effects between cyclists’ exposure to air and noise pollution and acute changes in heart rate variability among cyclists in Montreal, Québec. The aim of this study is to assess the impact of total inhaled dose of particulate matter and total noise exposure on the variations of electrocardiogram (ECG) parameters between pre-cycling and post-cycling periods. In other words, all other things being equal, what would be the impact of traffic-related air and noise pollution on the ability of cyclists to recover?

## 2. Materials and Methods

### 2.1. Study Design

**Figure 1:**
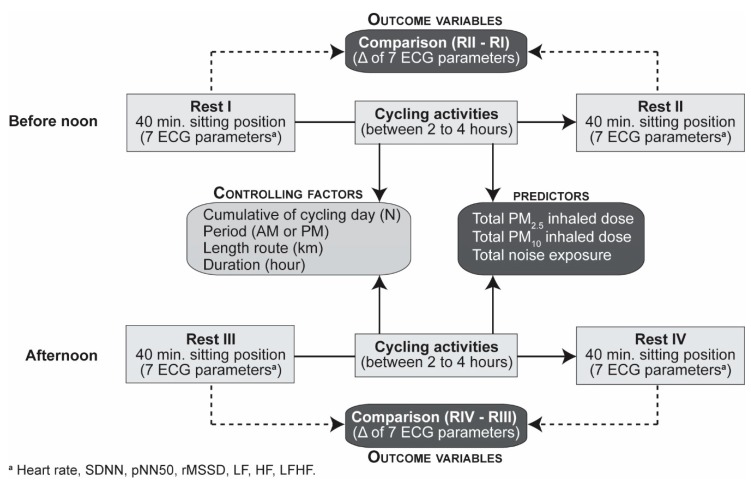
Evaluation framework. Electrocardiogram (ECG) parameters: heart rate (HR), standard deviation of the normal-to-normal intervals (SDNN), percentage of successive RR intervals that differ by more than 50 ms (pNN50), root mean square of successive RR interval differences; LF, low-frequency power (rMSSD), high-frequency power (HF), ratio of low- to high-frequency power (LF/HL). Particulate matter of less than 2.5 µm in diameter (PM_2.5_), particulate matter of less than 10 µm in diameter (PM_10_).

Four participants living in the Greater Montreal area were recruited at the Institut National de la Recherche Scientifique in Montreal to cycle for five days between 12 and 21 June 2019. We enlisted students at the Masters or Ph.D. levels. All participants were non-smokers and stopped taking coffee, alcohol, and intense exercises two weeks prior to the study. They had no previous cardiorespiratory medical history, such as asthma, stroke, angina, heart attack, or coronary heart disease. To minimize exposure to pollution, participants were scheduled to arrive before 7:00 in the morning because at that time there was lower air and noise exposure in Montreal. Once at the Research Centre, they were fitted with the Hexoskin smart shirt [21]. The evaluation framework consisted of four periods: a rest period before and after cycling, before noon and afternoon (Figure 1). Rest periods consisted of 40 min in a closed and quiet room (temperature 23 °C, humidity between 40 and 60 percent) to monitor continuous ECG tracings, and while cycling, we measured traffic-related air pollution and noise exposure. Our study analysis framework for heart rate variability is within similar evaluation frameworks that were done in previous studies [10,22]. Each participant cycled between 2 to 4 h before noon and afternoon. Travel distances were relatively similar amongst the participants during each cycling period, whereas the duration varied because of different traffic conditions and slopes encountered while cycling. During cycling activities, the participants were instructed to take a 5 min break after 40 min of cycling. Concretely, they cycled four to six routes.

We randomly divided the participants into two groups: two participants cycled with a mask and the two others without a mask. We would like to point out that we used the Techno Plus^TM^ Mask with Techno^TM^ Filter [23]. This study has been approved by the Institutional Review Board of The Institut National de la Recherche Scientifique (Project N°CER-15-391), and informed consent was obtained from each participant before the start of the study.

### 2.2. Air Pollution and Noise Exposure Estimation during Cycling Activity

Data collection used six types of devices: (1) Aeroqual Series 500 device, (2) Brüel and Kjaer personal noise dosimeter type 4448 (class 1), (3) Hexoskin Smart T-shirt, (4) Garmin watch Forerunner 920 XT, (5) Garmin VIRB XE, and (6) cell phone. With the exception of the Hexoskin Smart T-shirt and the GPS watch, all instruments were front-mounted near the handlebars for each participant. We measured real-time air pollution using the Aeroqual Series 500 with one sensor for particulate matter (PM_2.5_/PM_10_). It measured one-minute average concentration of air pollution in µg/m^3^, temperature in degree Celsius, and percentage of humidity within the cyclist’s surrounding environment. We then appraised the pollutants’ inhalation dose using the Hexoskin Smart T-shirt as it assesses the heart rate, breathing rate, VO_2_ max, and minute ventilation [14,24,25] while cycling. Thus, the pollutants’ inhalation dose per minute was obtained by multiplying the minute ventilation (VE) in liter per minute analyzed through the Hexoskin by air pollutant concentration value in µg/m^3^ acquired via the Aeroqual sensor [14] as follows:

(1) $$I=\left(VE\times 0.001\right)\times PM_{2.5}\;\mathrm{or}\;I=\left(VE*0.001\right)\times PM_{10}.$$

For each cycling activity (AM or PM), we then summed the inhaled dose per minute for PM_2.5_ and PM_10_ pollutants to obtain the cumulative inhaled doses (µg).

Noise exposure was assessed with class 1 dosimeter (Brüel and Kjaer personal noise dosimeter type 4448). This device measures average noise intensity for 1-min exposure. We are interested in the A-weighted equivalent continuous sound level which represents in decibel the average for 1-min time resolution (*L*_Aeq,1min_). We calibrated all dosimeters with the sound calibrator type 4231 to ensure accuracy daily. The noise dose is the cumulative exposure to noise over time. It is presented as a percentage of a reference dose, which enables us to calculate daily maximum acceptable dose. Classically, according to Berger [26], it is calculated as follows:

(2) $$D=\frac{100}{T_{c}}T_{i}10^{\left(\frac{L_{i}-L_{c}}{q}\right)},$$

where *D* is the total noise dose (in percentage), *T_c_* the criterion sound duration (e.g., 24 h), *T_i_* time exposure spent in the *i^th^* interval in hours, *L_c_* the criterion sound level (e.g., 53 dB(A)), *L_i_* the noise exposure intensity during the *i^th^* time interval and *q* the exchange rate parameter (dB)) (e.g., 10 for an exchange rate of 3 dB).

**Figure 2:**
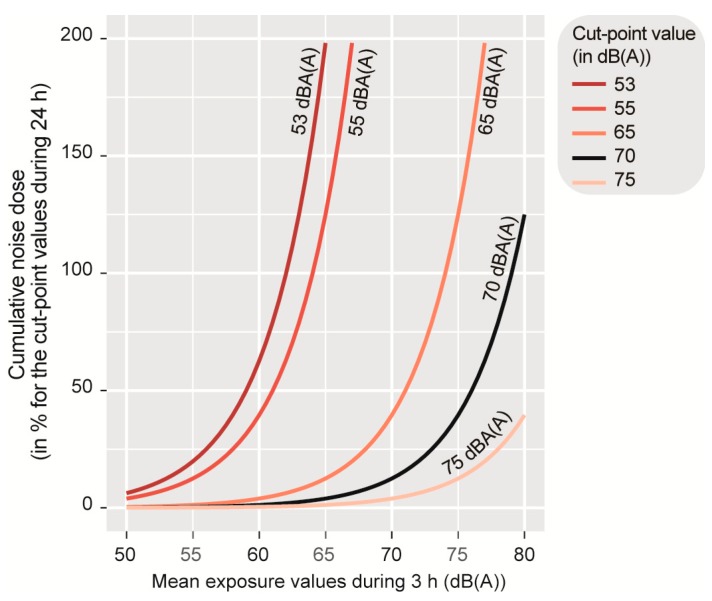
Estimated cumulative noise dose during 3 h by retained cutoffs.

On the basis of this formula, it is needed to select a cutoff value for the average noise exposure (*c*) and a time period (*h*). In a recent report, The World Health Organization recommends several guidelines values for the environmental noise exposure: 53 decibels (dB) during 24 h (*L*_den_) for the average road traffic noise because values above this cutoff are associated with adverse health effects, and 70 dB L_Aeq,24h_ for average leisure noise exposure. In Canada, provinces have, however, different thresholds. The Ontario Highway Traffic Act retains 55 dB(A), while the Quebec Transport Act considers 65 dB(A). It should be noted that participants cycled on average 3 h in the morning and 3 h in the afternoon. Variations in noise exposure doses are based on different thresholds (53, 55, 65, 70, 75 dB(A)), as shown in Figure 2. For instance, if the threshold of 53 dB(A) is used over 24 h then an average exposure of 60 dB(A) over three hours would be 53% compared with 198% for an average exposure of 65 dB(A). The thresholds of 53 and 55 dB(A) were not adopted because the noise doses calculated for our observations are much bigger. To a lesser extent, the same problem also arises for the 65 dB(A) threshold. The opposite problem arises with the threshold of 75 dB(A), the calculated doses are systematically too small and vary too little to be interpreted. Finally, the 70 dB(A) threshold offered the best compromise between calculated dose variability and meaningfulness of the measurement. Therefore, with the threshold of 70 dB(A), it can be assumed that adverse health effects will be more important, especially on heart function.

Furthermore, a Garmin watch Forerunner 920 XT was used to collect a global position system trace with one-second time resolution, while the Garmin VIRB XE videotaped the cyclist to probe conflict with adjacent traffic. In addition, the cell phone allowed to monitor live the cyclist’s activities through Life 360 and permitted them to follow a Google Maps route predetermined a-priori by the research team. Note here that each participant cycled specific routes that were never similar.

### 2.3. Heart Rate Variability Parameters

Hexoskin has built-in sensors in the smart t-shirt to record real-time cardiac signals. It identifies a heart wave sampling rate of 256 Hz, a heart rate between 30 and 220 beats per minute, a QRS complex, and an RR interval with a resolution of 4 ms [21]. Each participant was thus fitted with the Hexoskin Smart T-shirt for continuous monitoring of the electrocardiogram for 40 min in the sitting position. Electrocardiogram recordings started approximately with a 10-min period of preparation (i.e., wearing the cyclist’s equipment, plugging the Hexoskin T-shirt, etc.) after arrival and roughly a 30-min lunch prior to taking baseline measurement for the Rest I/Rest III, respectively. As for the Rest II/IV, each participant was directed immediately into the measurement room after cycling and arriving at the research center. A complete 40-min segment of the RR interval was exported from the Hexoskin platform to the R for statistical computing software [27] with the RHRV package [28].

Then, we extracted repeated 5 min RR intervals to process ECG parameters: heart rate (HR), standard deviation of the normal-to-normal intervals (SDNN), percentage of successive RR intervals that differ by more than 50 ms (pNN50), root mean square of successive RR interval differences (rMSSD), low-frequency power (LF: 0.04–0.15Hz), high-frequency power (HF: 0.15–0.40 Hz), and ratio of low- to high-frequency power (LF/HF). We collected a total of 255 5-min segments of heart rate variability parameters from the four participants after taking into account abnormal segments and missing periods. Different authors, hence, indicate that HF, rMSSD, and pNN50 reflect parasympathetic regulation and would be associated with changes in heart’s rhythm [8,9,17], whereas the SDNN represent global power [9,29].

On the other hand, LF reflects cardiovascular baroreceptor activity which reports information related to blood pressure and communicates this information directly to the central nervous system [30]. According to these authors, when there is excessive blood pressure, ‘baroreceptors inhibit the sympathetic branch by decreasing peripheral resistance, while activation of the parasympathetic branch induces bradycardia and arterial vasodilatation’ [30].

### 2.4. Statistical Analysis

All statistical analysis was performed using R for statistical computing software version 3.6.1 [27]. Several Bayesian linear models were built by using the *BRMS* package [31] to examine a potential association between air pollutants and noise exposure and ECG parameters. The outcome variables of the models were the variation difference within the 5 min measurement windows for ECG parameters between corresponding periods (Rest I versus Rest II, and Rest III, versus Rest IV). Next, we calculated cumulative inhaled doses for the two air pollutants (PM_2.5_, and PM_10_), and the cumulative dose for noise exposure, before noon and afternoon periods for each participant. These cumulative doses were introduced as predictors in the models. We included a priori five controlling factors: (1) Cumulative of cycling days (*n* = 1 to 5), (2) period of day (dummy variable: AM or PM), (3) distance cycled during the period (kilometers), (4) duration of the cycling activity (hours), (5) number of repeated 5 min RR intervals for the rest period (*n* = 1 to 8). We expected that the first four confounding factors could increase the participant’s fatigue, which could have an impact on the variation of the ECG parameters. Moreover, we should also anticipate higher levels of heart rate variability (HRV) parameters and lower levels of heart rate for Rest I compared to Rest II, Rest III, and Rest IV. Note that the fifth controlling factor—number of repeated 5 min RR intervals—was introduced as a temporal autocorrelation term (moving average, MA = 1). Finally, we introduced an interaction term between each cumulative dose and the participant to estimate participants’ specific responses to the pollutants. Thus, this allowed us to compare the responses between the participants with and without a mask.

In short, we built 21 separate Bayesian regression models for three predictors and seven ECG outcomes adjusting for possible confounders, while the final model included all predictor variables. We fitted our models using four chains, each with 4000 iterations where the first 1000 were used as a warmup for sampling [31]. Samples were implemented using the No-U-turn Sampler (NUTS), which is an extension of the Hamiltonian Monte Carlo Method, to increase the effectiveness in performing tasks related to the optimal number in each iteration [32]. We expected levels of air pollutant inhalation and noise exposure to increase heart rate, thereby reducing heart rate variability parameters. Indeed, the HRV of those who cycled with a mask should be higher than those who cycled without masks.

Finally, we applied the Savage–Dickey density ratio to compute the Bayes factor of different parameters for the separated models and the full model [33]. The Bayes factor was used to examine the level by which the posterior distribution reflects or not the null hypothesis, thereby probing if the impact was really significant or not [34]. We indeed measured the Bayesian parameters using the Bayesian applied regression through *rstanarm* package based on prior and posterior distributions [35]. The interpretation of the Bayes factor can be found elsewhere within the scientific literature [36]. Bayes factors are ratios that compare the odds of observed data fitting under the null hypothesis with the odds of fitting under the alternative hypothesis [37]. It represents the probability of the likelihood of the observed data given the null hypothesis by the likelihood of the observed data given the alternative hypothesis [37]. Given the above, as the Bayes factor increases, evidence strengthens the null hypothesis, while the inverse yields the opposite, which supports the alternative hypothesis.

## 3. Results

### 3.1. Descriptive Statistics

Four participants, three males and one female, participated in the study over five days in mid-June 2019. They were aged 25–37 years with a height of 163 to 175 cm, a weight of 52 to 76.5 kg and a body mass index of 19.6–25 kg m^−2^. Table 1 shows the participants’ cardiovascular characteristics prior to cycling. These obtained values are comparable with other baseline measures in previous studies [38,39].

For each period (AM or PM), participants cycled an average of 41.7 km (range: 30.7–55.8) during 3 h (range: 2.2–4.3) with an average speed of 13.8 km/h (11.0–16.3 km/h) (Table 2). The latter also shows the level of exposure during the cycling activity period. On average, the exposure to PM_2.5_ and PM_10_ were 4.1 µg/m^3^ (range: 1.8–10.7) and 13.7 µg/m^3^ (range: 6.9–36.4) respectively, and 71.9 dB(A) (range: 68.9–73.7) for the noise. The total inhaled doses for particulate matter were 32.1 µg of PM_2.5_ (range: 1.0–116.3) and 107.1 µg of PM_10_ (range: 3.3–370.3). For the noise dose, the percentage of a 70 dB(A) cutoff value during 24 h was 20.5% on average (range: 9.0 to 36.6).

Table 3 presents descriptive statistics for variations of the ECG parameters before and after the cycling activity (Rest II minus Rest I, and Rest IV, Rest III, see Figure 1). We observed an increase in the heart rate (mean: 6.0, SD: 12.6) and the ratio LFHF (mean: 0.2, SD: 2.6). Inversely, all the other ECG parameters decreased: SDNN (mean: −17.9, SD: 37.1), pNN50 (mean: −4.9, SD: 17.7), rMSSD (mean: −11.2, SD: 36.4), LF (mean: −400, SD: 1113), HF (mean: −201, SD: 855).

### 3.2. Correlation Between ECG Parameters, Particulate Matter and Noise Measures

The Table 4 represents the correlation among variations of the cardiovascular parameters. Not surprisingly, the variation of rMSSD is highly and positively correlated with the variation of SDNN (r = 0.87), pNN50 (r = 0.71), LF (r = 0.76), and HF (r = 0.84). LF and HL are also highly and positively correlated (r = 0.75). The heart rate is negatively correlated with the other HRV parameters, in particular with the pNN50 (r = −0.75).

Concerning air and noise exposure, PM_2.5_ and PM_10_ are extremely correlated between them (r = 0.98), and we found a mildly positive correlation between the total dose of dB(A) and the total inhaled doses of PM_2.5_ (r = 0.31) and PM_10_ (r = 0.33). Due to a very high correlation rate between PM_2.5_ and PM_10_, we only considered PM_2.5_ for our subsequent analysis. We also chose the total inhaled dose of PM_2.5_ because, in a very schematic way, PM_2.5_ and much smaller particles, such as PM_1,_ are the most biotoxic since they can penetrate deep into the lungs and lung alveoli [40,41], thereby predicting health risks from anthropogenic particulate matter much better than PM_10_ [42].

### 3.3. Effects of Particulate Matter and Noise Assessed Separately

Table 5 shows associations between PM_2.5_ inhaled doses while cycling and variations in ECG parameters. Recall that the Bayes factor (BF) is interpreted as follows: a Bayes factor greater than 1 makes a proof judgment in favor of the null hypothesis or the numerator model, whereas when it is less than 1, it rules in favor of the alternative hypothesis or the denominator model. We found in the literature various thresholds of the BF for interpreting effect size. Jeffreys interprets Bayes factors as follows: (1) bf = 1–3: Anecdotal, (2) bf = 3–10: Moderate, (3) bf = 10–30: Strong, (4) bf = 30–100: Very strong, and (5) bf > 100: Extreme [36]. As for the interpretation of Raftery which is more recent, it states that: (1) bf = 1–3 is weak, (2) bf = 3–20 is positive, (3) bf = 20–150 is strong, and (4) bf > 150 is very strong [43].

We observed that per 1 µg increase in cumulative inhaled dose of PM_2.5_ was associated with 0.48 (95% credible interval (CI): 0.22; 15.61) increase in variation of heart rate between corresponding periods. Given the study data, the presence of the effect is 13.78 times more likely under the alternative hypothesis than no effect at all. The interaction coefficient (dose*participant) allowed us to examine whether or not wearing a mask had an impact or not. Let us recall here that the first and second participants did not wear pollution masks, while the third and fourth participants cycled with masks. It is also worth mentioning that our reference comparator is the first participant. In comparison to the benchmark, per 1 µg increase in cumulative PM_2.5_ inhaled dose was negatively associated with −0.34 (95% CI: −0.60; −0.10) and −0.36 (95% CI: −0.62; −0.10) decline in heart rate variations for the third and fourth participants (with a mask) with Bayes factors of 1.75 and 1.61, respectively. As shown here, these Bayes factors are nonetheless very low for these interaction coefficients.

For the other parameters, only three other coefficients appeared to be significant: 1 µg in cumulative PM_2.5_ inhaled dose increase was associated with −0.76 (95% CI: −1.50; −0.03) decrease in ∆SDNN, −0.41 (95% CI: −0.73; −0.09) decline in ∆pNN50 and 0.80 (95% CI: 0.10; 1.50) increase for the interaction between inhaled dose of PM_2.5_ with the third participant and ΔSDNN. Observed data provided weak or anecdotal evidence against the null hypothesis for the ∆pNN50 with a Bayes factor equal to 1.29, whilst coefficients were three times more likely true within the null hypothesis than the alternative hypothesis for the ∆SDNN.

Table 6 conveys that an increment of one percent in total noise dose was associated with 0.49 (95% CI: 0.17; 0.83) increase in variation of the heart rate. We noted, according to the Bayes factor, that this association was 5.04 times more likely outside the null hypothesis. We, consequently, detected that it was negatively associated with the interaction coefficient (Noise dose*participant). There were, as consequence, −0.59 (95% CI: −1.02; −0.15) and −0.49 (95% CI: −0.85; −0.12) declines in heart rate variations for the second and third participants, respectively. The observed data were 3.17 times and 2.10 times more probable to occur under the alternative hypothesis compared to the null hypothesis for the aforementioned participants. Nonetheless, there is an anecdotal posterior probability that this is true under the sun. Other parameters were not significant either except their interaction coefficients for the second and third participants that asymptotically depicted evidence in favor of the null hypothesis according to their Bayes factors.

### 3.4. Effects of Particulate Matter and Noise Assessed Simultaneously

The inhaled PM_2.5_ dose and noise dose have been shown to have a small impact only on heart rate, as demonstrated previously. Are these effects combined and attempt to show that multi-exposure (air pollution and noise exposure) might increase short-term health impacts? We likewise examined whether there was a combined effect of these two exposures on electrocardiogram parameters. However, the results reported in Table 7 show that the noise dose no longer has an effect once the inhaled PM_2.5_ dose is introduced. The coefficients of the latter, on the other hand, which were significant previously, remain unchanged.

## 4. Discussion

To our best knowledge, this is the first study to report the short-term impact of simultaneous traffic-related particulate matter with aerodynamic diameters of less than 2.5µm (PM_2.5_) and noise exposure on cardiac function among healthy young cyclists in Montreal, Canada. It is important to remember that the metric of exposure used in statistical analysis was the 5-min RR intervals within which we analyzed corresponding electrocardiogram parameters and cumulative inhaled doses for air pollution and noise dose exposure. Our hypothesis was that short-term exposure to air and noise pollution would lead to the reduction of heart rate variability due to the dysfunction of the autonomic nervous system. We found only limited effects on the heart rate variation. These effects are, however, so low that could explain the lack of effects for other parameters related to heart rate variability. This is consistent with the results of previous studies which show that although exposure to PM_2.5_ may increase heart rate, this does not translate into changes in the parameters of heart rate variability in low polluted environments [44]. Different authors found no associations between PM_2.5_ and heart rate variability parameters in their systematic review, regardless of small negative associations across all parameters, especially because of large confidence intervals that comprised sometimes the null effect [7]. Moreover, Dzhambov and Dimitrova in a recent systematic review [45], observed a weak association between traffic-related noise exposure and blood pressure in children, but that was wiped out after considering the soundness of included studies. It is also known that ‘in low traffic-related air pollution (TRAP) environments, intermittent physical activity has stronger beneficial effects on systolic blood pressure than in high-TRAP environments’ [4]. These results should, nevertheless, be read with caution so as not to minimize the potential impact of exposure to noise and air pollution on health, especially since there are various studies which have found their impact on the health of the population [46].

The levels of air pollution and noise exposure are probably insufficient in Montreal to show significant and important effects between exposure to pollutants and absolute variations in heart rate variability. For instance, Montreal had an average air quality index of 6.8 and 7 µg/m^3^ for PM_2.5_ in 2017 and 2018, respectively [47]. These levels are within the World Health Organization target for the PM_2.5_ to ensure no adverse health effects. For this reason, studies should be carried out in the world’s most polluted cities, especially in South East Asian countries or where there is more unavailable data, like in some parts of Africa. By the way of comparison for Montreal, the city of Gurugram in India, which is the most polluted city in the world, had 145.6 and 135.8 µg/m^3^, while New Delhi had 108.26 and 113.5 µg/m^3^ for the PM_2.5_ in 2017 and 2018, respectively [47]. Without prejudice, a study under these conditions will be more likely to demonstrate the impact of air pollution and noise exposure on electrocardiogram parameters.

Let us recall that participants cycled, on average, 3 h in the morning and 3 h in the afternoon. As utilitarian cyclists usually commute between 30 and 60 min, it can be assumed that cycling in Montreal for utilitarian trips has no short-term or at least meager adverse health effects. This could lead to the adoption of a healthy lifestyle, such as active transportation, thereby promoting physical activity that could contribute to reducing and prevent chronic diseases. These results are consistent with current studies indicating that the benefits of cycling outweigh the risks of air pollution to the health of the population in urban centers [13]. These authors found that years of life expectancy gained through cycling were much larger than in those who commuted by car or public transport, such as bus, train, and subway, because of increased physical activity despite higher inhaled doses. Besides, physical activity has a positive impact on reducing the inflammatory processes. Hence, its increase would prevent cardiovascular risk factors [4]. As a result, a built environment conducive to physical activity while cycling could improve the physical health and well-being of urban residents [48].

Keep in mind, however, that the exposure of PM_2.5_ may considerably fluctuate according to space and time of the day. It is, nonetheless, difficult to assess its long-term impact with empirical data at least on cyclists because of practicability and associated cost. The long-term impacts are probably very detrimental to health, as has been stipulated by different authors [49,50]. Since this impact is difficult to assess in the long run, cycling facilities, such as bicycle paths, should reduce air pollution and noise exposure by moving them away from moving vehicles. Municipal planners could accommodate whenever possible these cycle ways in green or residential areas. They should thus foster the establishment of cycling infrastructure to reduce inhaled or uptake doses through spatial separation of cyclists from high traffic roads [51]. Ramos et al. stipulate that these facilities ‘should be achieved with separated bicycle facilities, low volume routes, and off-peak travel’ [52].

This study, therefore, offers us an estimate of simultaneous exposure to particulate matter with a diameter less than 2.5 µm (PM_2.5_) and noise exposure on cyclists’ cardiac function. There are, however, some strengths and limitations, as in any research project. Without being exhaustive, one of the strengths is that it has examined pollutants’ exposure at the individual level, particularly under real-world conditions. It also shows in detail the measurements of air pollution, noise exposure, and electrocardiogram parameters, as well as possible confounding factors deemed important. Indeed, the study design allowed us to disentangle the influence of wearing mask or not. As limits, air pollution and noise exposure have higher spatial and temporal distribution, which can increase the risk of bias. Although this may be a limit, the distances and routes traveled by the participants encompass the distribution within the Montreal Island. In addition, the small sample size may fail to measure the effect of air pollution and noise exposure on the cardiac function when it exists, while the temporality of the evaluation does not make it possible to examine the additive effects with long-term consequences. This demonstrates the need for further large-scale studies to generalize on the health and well-being of the population.

## 5. Conclusions

This study’s findings support that separate exposure to PM_2.5_ and noise exposure related to road-traffic lead to a minor increase in heart rate variation between corresponding pre-cycling and post-cycling periods. When considering the simultaneous effect in relation to the aforementioned pollutants, that effect disappears for noise exposure, however. These results should not be extrapolated to the general public, such as commuters or cyclists as such, because of small sample size, albeit they show the absence of effects on heart rate variability for the participants. Given that it is based on a rigorous methodology that can grasp short-term health effects arising from multi-exposure related to traffic exposure, it might be interesting to examine the synergetic effects of multiple exposures, such as traffic-related air pollutants and noise exposure on cardiac function. This would better position the use of bicycles across the city while reducing exposure to pollutants both from traffic-related air pollution and from noise exposure, thereby shedding light on their health impacts.

## Figures and Tables

**Table 1:** Baseline descriptive cardiovascular measurements ^1^.

ECG Parameters	Mean	SD	Min	P25	P50	P75	Max	IQR
HR (bpm)	69	11	49	61	69	77	104	16
SDNN (msec)	100	47	43	76	94	109	341	34
pNN50 (%)	40.2	23.1	1.1	24.6	32.0	56.8	84.1	32
rMSSD (msec)	83	52	18	47	60	118	306	71
LF (msec^2^)	1389	1418	147	609	923	1704	8842	1095
HF (msec^2^)	1163	1169	79	352	610	1877	5923	1525
LF/HF	2.11	1.60	0.08	0.81	2.04	2.96	8.64	2.15

^1^ Obtained from the Rest I for the five days, four participants and eight lags of 5 min. Electrocardiogram (ECG) parameters: heart rate (HR), standard deviation of the normal-to-normal intervals (SDNN), percentage of successive RR intervals that differ by more than 50 ms (pNN50), root mean square of successive RR interval differences; LF, low-frequency power (rMSSD), high-frequency power (HF), ratio of low- to high-frequency power (LF/HL).

**Table 2:** Descriptive statistics of air/noise pollution exposure and inhaled doses while cycling.

Characteristics	Mean	SD	Min	P25	P50	P75	Max	IQR
Cycling activity (AM or PM)								
Length (km)	41.7	5.3	30.7	38.3	41.3	44.2	55.8	6.0
Duration (h)	3.0	0.4	2.2	2.8	3.0	3.3	4.3	0.5
Speed (km/h)	13.8	1.2	11.0	13.1	13.7	14.6	16.3	1.5
Air pollution and noise (AM or PM)								
PM_2.5_ (µg/m^3^)	4.1	2.1	1.8	2.6	3.6	4.9	10.7	2.3
PM_10_ (µg/m^3^)	13.7	6.8	6.9	9.3	11.8	15.0	36.4	5.7
Noise (*L*_Aeq,1min_)	71.9	1.2	68.9	71.3	72.0	72.6	73.7	1.3
Exposure (AM or PM)								
Total inhaled dose of PM_2.5_ (µg)	32.1	30.8	1.0	12.0	18.6	38.3	116.3	26.2
Total inhaled dose of PM_10_ (µg)	107.1	99.9	3.3	44.4	78.8	120.9	370.3	76.6
Total dose of noise (%)^1^	20.5	6.8	9.0	15.0	19.9	24.1	36.6	9.1

PM_2.5_: Particulate matter of less than 2.5 µm in diameter, PM_10_: Particulate matter of less than 10 µm in diameter. *L*_Aeq,1min_: Average noise intensity for 1-minute exposure in decibels (dB(A)). ^1^ Total noise dose in percentage calculated with the cutoff of 70 dB during 24h (*L*_Aeq,24h_).

**Table 3:** Descriptive statistics of the variation difference in 5 min window of ECG parameters ^1^.

ECG Parameters	Mean	SD	Min	P25	P50	P75	Max	IQR
ΔHR (bpm)	6.0	12.6	−43.0	−2.4	3.8	14.1	42.6	16.4
ΔSDNN (msec)	−17.9	37.1	−244.4	−36.0	−11.1	4.0	92.8	40.0
ΔpNN50 (%)	−4.9	17.7	−60.4	−14.4	−2.6	2.2	53.3	16.6
ΔrMSSD (msec)	−11.2	36.4	−169.5	−28.1	−8.8	8.2	126.9	36.3
ΔLF (msec)	−400.5	1112.8	−7400.1	−618.1	194.2	107.4	4714.3	725.6
ΔHF (msec)	−201.0	854.6	−4592.0	−411.3	−60.1	99.9	3211.9	511.2
ΔLF/HF	0.2	2.6	−10.9	−08	0.1	1.8	7.9	2.6

^1^ Obtained from the AM and PM variations (Rest II minus Rest I, Rest III minus Rest IV) for five days, four participants, and eight lags of 5 min. Variations in electrocardiogram (ECG) parameters. ΔHR: Variation in heart rate, ΔSDNN: Variation in standard deviation of the normal-to-normal intervals, ΔpNN50: Variation in percentage of successive RR intervals that differ by more than 50 ms, ΔrMSSD: Variation in root mean square of successive RR interval differences, ΔLF: Variation in low-frequency power, ΔHF: Variation in high-frequency power, ΔLF/HL: Variation in ratio of low- to high-frequency power.

**Table 4:** Pearson correlation between the variations of the cardiovascular measurements.

ECG Parameters	ΔHR	ΔSDNN	ΔpNN50	ΔrMSSD	ΔLF	ΔHF
ΔSDNN	−0.418 ***					
ΔpNN50	−0.750 ***	0.708 ***				
ΔrMSSD	−0.358 ***	0.873 ***	0.704 ***			
ΔLF	−0.199	0.697 ***	0.500 ***	0.757 ***		
ΔHF	−0.176 *	0.667 ***	0.481 ***	0.838 ***	0.746 ***	
ΔLF/HF	0.471 ***	−0.409 ***	−0.482 ***	−0.388 ***	−0.099	−0.268 **

Sign: * 0.05, ** 0.01, *** 0.001. Electrocardiogram (ECG) parameters. ΔHR: Variation in heart rate, ΔSDNN: Variation in standard deviation of the normal-to-normal intervals, ΔpNN50: Variation in percentage of successive RR intervals that differ by more than 50 ms, ΔrMSSD: Variation in root mean square of successive RR interval differences, ΔLF: Variation in low-frequency power, ΔHF: Variation in high-frequency power, ΔLF/HL: Variation in ratio of low- to high-frequency power.

**Table 5:** Effects of total inhaled doses of PM_2.5_ on ECG parameters variability ^1^.

	**ΔHR ^1^**	**BF ^2^**	**ΔSDNN ^1^**	**BF ^2^**	**ΔpNN50 ^1^**	**BF ^2^**
PM_2.5_ dose	0.48 (0.22; 15.61)	13.78	−0.76 (−1.50; −0.03)	0.32	−0.41 (−0.73; −0.09)	1.29
PM_2.5_ dose: P.2 ^3^	−0.22 (−0.58; 0.14)	0.13	−0.10 (−1.13; 0.93)	0.04	0.23 (−0.20; 0.66)	0.13
PM_2.5_ dose: P.3 ^3^	−0.34 (−0.60; −0.10)	1.75	0.80 (0.10; 1.50)	0.30	0.29 (−0.02; 0.59)	0.29
PM_2.5_ dose: P.4 ^3^	−0.36 (−0.62; −0.10)	1.61	0.48 (−0.24; 1.12)	0.06	0.15 (−0.17; 0.47)	0.08
	**ΔrMSSD ^1^**	**BF ^2^**	**ΔLF ^1^**	**BF ^2^**	**ΔHF ^1^**	**BF ^2^**
PM_2.5_ dose	−0.43 (−1.12; 0.30)	0.13	−4.59 (−25.12; 0.11)	0.12	4.03 (−10.35; 18.29)	0.29
PM_2.5_ dose: P.2 ^3^	−0.51 (−1.45; 0.38)	0.08	−9.31 (−34.76; 16.11)	0.11	−14.95 (−31.24; 1.19)	1.33
PM_2.5_ dose: P.3 ^3^	0.49 (−0.18; 1.15)	0.10	8.69 (−9.32; 26.66)	0.09	2.23 (−9.98; 14.78)	0.23
PM_2.5_ dose: P.4 ^3^	0.30 (−0.38; 1.00)	0.05	−3.01 (−23.15; 16.15)	0.07	0.22 (−13.71; 14.31)	0.24
	**ΔLF/HF ^1^**	**BF ^2^**				
PM_2.5_ dose	0.05 (−0.04; 0.13)	0.03				
PM_2.5_ dose: P.2 ^3^	0.00 (−0.12; 0.13)	0.02				
PM_2.5_ dose: P.3 ^3^	−0.04 (−0.12; 0.05)	0.02				
PM_2.5_ dose: P.4 ^3^	0.01 (−0.08; 0.09)	0.01				

Electrocardiogram (ECG) parameters. ΔHR: variation in heart rate; ΔSDNN: Variation in standard deviation of the normal-to-normal intervals, ΔpNN50: Variation in percentage of successive RR intervals that differ by more than 50 ms, ΔrMSSD: Variation in root mean square of successive RR interval differences, ΔLF: Variation in low-frequency power, ΔHF: Variation in high-frequency power, ΔLF/HL: Variation in ratio of low- to high-frequency power. PM_2.5_: Particulate matter of less than 2.5 µm in diameter. ^1^ Estimate and 95% CI adjusted for cumulative of cycling days (*n* = 1 to 5), period of day (AM or PM), distance cycled during the period (kilometers), duration of the cycling activity (hours), number of repeated 5 min RR intervals for the rest period (*n* = 1 to 8), and the participant. ^2^ Bayes factor (BF) versus 0. ^3^ Interaction between total inhaled dose of PM_2.5_ and participants 2 to 4 in comparison with the participant 1 as reference. Note participants 1 and 2 did not wear a pollution mask, while participants 3 and 4 did.

**Table 6:** Effects of total noise dose exposure on ECG parameters variability ^1^.

	**ΔHR ^1^**	**BF ^2^**	**ΔSDNN ^1^**	**BF ^2^**	**ΔpNN50 ^1^**	**BF ^2^**
Noise dose	0.49 (0.17; 0.83)	5.04	−0.30 (−1.50; 0.94)	0.08	−0.35 (−0.82; 0.13)	0.23
Noise dose: P.2 ^3^	−0.59 (−1.02; −0.15)	3.17	1.11 (−0.26; 2.47)	0.18	0.73 (0.23; 1.21)	4.93
Noise dose: P.3 ^3^	−0.49 (−0.85; −0.12)	2.10	1.32 (0.07; 2.53)	0.35	0.51 (0.07; 0.96)	1.10
Noise dose: P.4 ^3^	−0.18 (−0.62; 0.26)	0.11	0.30 (−1.17; 1.79)	0.05	−0.05 (−0.56; 0.47)	0.09
	**ΔrMSSD ^1^**	**BF ^2^**	**ΔLF ^1^**	**BF^2^**	**ΔHF^1^**	**BF ^2^**
Noise dose	−0.05 (−1.23; 1.08)	0.10	−0.21 (−31.8; 31.6)	0.19	3.81 (−20.10; 27.46)	0.40
Noise dose: P.2 ^3^	1.53 (0.46; 2.57)	2.77	15.10 (−8.3; 38.4)	0.19	18.41 (2.67; 33.99)	3.71
Noise dose: P.3 ^3^	0.61 (−0.37; 1.62)	0.10	9.32 (−13.2; 31.7)	0.11	8.35 (−6.74; 23.35)	0.47
Noise dose: P.4 ^3^	0.03 (−1.10; 1.15)	0.06	5.02 (−19.8; 30.0)	0.09	1.56 (−15.77; 18.87)	0.30
	**ΔLF/HF ^1^**	**BF ^2^**				
Noise dose	0.08 (−0.03; 0.18)	0.05				
Noise dose: P.2 ^3^	−0.07 (−0.31; 0.17)	0.05				
Noise dose: P.3 ^3^	−0.21 (−0.35; 0.07)	1.70				
Noise dose: P.4 ^3^	−0.15 (−0.36; 0.07)	0.09				

Electrocardiogram (ECG) parameters. ΔHR: Variation in heart rate, ΔSDNN: Variation in standard deviation of the normal-to-normal intervals, ΔpNN50: Variation in percentage of successive RR intervals that differ by more than 50 ms, ΔrMSSD: Variation in root mean square of successive RR interval differences, ΔLF: Variation in low-frequency power, ΔHF: Variation in high-frequency power, ΔLF/HL: Variation in ratio of low- to high-frequency power. Noise dose in percentage calculated with the cutoff of 70 dB during 24h (*L*_Aeq,24h_). ^1^ Estimate and 95% CI adjusted for cumulative of cycling days (*n* = 1 to 5), period of day (AM or PM), distance cycled during the period (kilometers), duration of the cycling activity (hours), number of repeated 5 min RR intervals for the rest period (*n* = 1 to 8), and the participant. ^2^ Bayes factor (BF) versus 0. ^3^ Interaction between total dose of dB(A) and participants 2 to 4 in comparison with the participant 1 as reference. Note participants 1 and 2 did not wear a pollution mask, and participants 3 and 4 did.

**Table 7:** Effects of total inhaled doses of PM_2.5_ and total noise dose exposure levels on ECG parameters variability ^1^.

	**ΔHR ^1^**	**BF ^2^**	**ΔSDNN ^1^**	**BF ^2^**	**ΔpNN50 ^1^**	**BF ^2^**
PM_2.5_ dose	0.40 (0.16; 0.66)	8.12	−0.43 (−1.13; 0.25)	0.08	−0.23 (−0.53; 0.05)	0.17
Noise dose	0.28 (−0.20; 0.76)	0.16	0.90 (−0.43; 2.29)	0.18	0.12 (−0.47; 0.69)	0.11
PM_2.5_ dose: P.2 ^3^	−1.75 (−2.57; −0.94)	238.42	−0.21 −2.51; 2.10)	0.13	1.28 (0.35; 2.20)	5.97
PM_2.5_ dose: P.3 ^3^	−0.35 (−0.60; −0.11)	2.44	0.50 (−0.17; 1.19)	0.11	0.22 (−0.05; 0.51)	0.16
PM_2.5_ dose: P.4 ^3^	−0.37 (−0.62; −0.13)	4.08	0.27 (−0.40; 0.95)	0.05	0.10 (−0.18; 0.37)	0.06
Noise dose: P.2 ^4^	−0.05 (−0.22; 0.13)	0.99	1.11 (−0.26; 2.47)	1.04	0.05 (−0.13; 0.23)	1.03
Noise dose: P.3 ^4^	−0.07 (−0.25; 0.10)	1.26	1.32 (0.07; 2.53)	0.83	0.04 (−0.14; 0.23)	1.03
Noise dose: P.4 ^4^	0.02 (−0.17; 0.20)	0.94	0.30 (−1.17; 1.79)	0.95	−0.02 (−0.21; 0.16)	0.99
	**ΔrMSSD ^1^**	**BF ^2^**	**ΔLF ^1^**	**BF ^2^**	**ΔHF ^1^**	**BF ^2^**
PM_2.5_ dose	−0.59 (−1.27; 0.08)	0.27	−4.41 (−24.52; 15.90)	0.12	−11.86 (−26.29; 2.67)	0.41
Noise dose	0.92 (−0.43; 2.22)	0.29	4.90 (−34.22; 43.92)	0.23	10.81 (−19.08; 39.75)	0.32
PM_2.5_ dose: P.2 ^3^	−2.6 (−4.28; −0.06)	1.40	−14.00 (−75.88; 46.21)	0.38	−96.37 (−140.6; −52.7)	921.5
PM_2.5_ dose: P.3 ^3^	0.58 (−0.06; 1.22)	0.29	9.45 (−9.81; 28.87)	0.17	11.98 (−1.80; 25.63)	0.49
PM_2.5_ dose: P.4 ^3^	0.24 (−0.38; 0.88)	0.07	−4.29 (−23.92; 15.22)	0.12	3.56 (−10.51; 17.23)	0.13
Noise dose: P.2 ^4^	0.20 (−0.16; 0.58)	1.64	5.83 (−9.34; 21.41)	1.12	8.61 (−1.90; 19.10)	3.38
Noise dose: P.3 ^4^	−0.09 (−0.46; 0.29)	1.10	−3.75 (−18.62; 11.39)	0.94	−3.76 (−14.14; 6.49)	1.11
Noise dose: P.4 ^4^	−0.01 (−0.38; 0.37)	0.97	−0.08 (−15.97; 15.96)	0.89	−0.14 (−11.15; 10.85)	0.97
	**ΔLF/HF ^1^**	**BF ^2^**				
PM_2.5_ dose	0.00 (−0.09; 0.09)	0.02				
Noise dose	−0.11 (−0.27; 0.06)	0.06				
PM_2.5_ dose: P.2 ^3^	−0.18 (−0.47; 0.13)	0.10				
PM_2.5_ dose: P.3 ^3^	0.01 (−0.08; 0.10)	0.02				
PM_2.5_ dose: P.4 ^3^	0.03 (−0.05; 0.12)	0.02				
Noise dose: P.2 ^4^	0.01 (−0.14; 0.16)	0.83				
Noise dose: P.3 ^4^	−0.05 (−0.18; 0.08)	0.88				
Noise dose: P.4 ^4^	−0.06 (−0.22; 0.11)	1.04				

Electrocardiogram (ECG) parameters. ΔHR: Variation in heart rate, ΔSDNN: Variation in standard deviation of the normal-to-normal intervals, ΔpNN50: Variation in percentage of successive RR intervals that differ by more than 50 ms, ΔrMSSD: Variation in root mean square of successive RR interval differences, ΔLF: Variation in low-frequency power, ΔHF: Variation in high-frequency power, ΔLF/HL: Variation in ratio of low- to high-frequency power. PM_2.5_: Particulate matter of less than 2.5 µm in diameter. Noise dose in percentage calculated with the cutoff of 70 dB during 24h (*L*_Aeq,24h_). ^1^ Estimate and 95% CI adjusted for cumulative of cycling day (*n* = 1 to 5), period of day (AM or PM), distance cycled during the period (kilometres), duration of the cycling activity (hours), number of repeated 5 min RR intervals for the rest period (*n* = 1 to 8), and the participant. ^2^ Bayes factor (BF) versus 0. ^3^ Interaction between total inhaled of PM_2.5_ and participants 2 to 4 in comparison with the participant 1 as reference. ^4^ Interaction between total dose of dB(A) and participants 2 to 4 in comparison with the participant 1 as reference. Note participants 1 and 2 did not wear a pollution mask, and participants 3 and 4 did.
